# Successive Respiratory Syncytial Virus Epidemics in Local Populations Arise from Multiple Variant Introductions, Providing Insights into Virus Persistence

**DOI:** 10.1128/JVI.01972-15

**Published:** 2015-09-09

**Authors:** Charles N. Agoti, James R. Otieno, Mwanajuma Ngama, Alexander G. Mwihuri, Graham F. Medley, Patricia A. Cane, D. James Nokes

**Affiliations:** a Epidemiology and Demography Department, Kenya Medical Research Institute—Wellcome Trust Research Programme, Kilifi, Kenya; b Department of Biomedical Sciences, Pwani University, Kilifi, Kenya; c Department of Global Health & Development, London School of Hygiene and Tropical Medicine, London, United Kingdom; d Public Health England, Salisbury, United Kingdom; e School of Life Sciences and WIDER, University of Warwick, Coventry, United Kingdom

## Abstract

Respiratory syncytial virus (RSV) is a global respiratory pathogen of humans, with infection occurring characteristically as recurrent seasonal epidemics. Unlike influenza viruses, little attention has been paid to the mechanism underlying worldwide spread and persistence of RSV and how this may be discerned through an improved understanding of the introduction and persistence of RSV in local communities. We analyzed 651 attachment (G) glycoprotein nucleotide sequences of RSV B collected over 11 epidemics (2002 to 2012) in Kilifi, Kenya, and contemporaneous data collected elsewhere in Kenya and 18 other countries worldwide (2002 to 2012). Based on phylogeny, genetic distance and clustering patterns, we set out pragmatic criteria to classify local viruses into distinct genotypes and variants, identifying those newly introduced and those locally persisting. Three genotypes were identified in the Kilifi data set: BA (*n* = 500), SAB1 (*n* = 148), and SAB4 (*n* = 3). Recurrent RSV epidemics in the local population were composed of numerous genetic variants, most of which have been newly introduced rather than persisting in the location from season to season. Global comparison revealed that (i) most Kilifi variants do not cluster closely with strains from outside Kenya, (ii) some Kilifi variants were closely related to those observed outside Kenya (mostly Western Europe), and (iii) many variants were circulating elsewhere but were never detected in Kilifi. These results are consistent with the hypothesis that year-to-year presence of RSV at the local level (i.e., Kilifi) is achieved primarily, but not exclusively, through introductions from a pool of variants that are geographically restricted (i.e., to Kenya or to the region) rather than global.

**IMPORTANCE** The mechanism by which RSV persists and reinvades local populations is poorly understood. We investigated this by studying the temporal patterns of RSV variants in a rural setting in tropical Africa and comparing these variants with contemporaneous variants circulating in other countries. We found that periodic seasonal RSV transmission at the local level appears to require regular new introductions of variants. However, importantly, the evidence suggests that the source of new variants is mostly geographically restricted, and we hypothesize that year-to-year RSV persistence is at the country level rather than the global level. This has implications for control.

## INTRODUCTION

Respiratory syncytial virus (RSV) is a leading viral cause of severe acute respiratory illness in human populations worldwide. Each year, the virus is responsible for ∼3 million hospitalizations and >50,000 deaths in individuals under 5 years old alone (1). RSV infections are recognized to be highly seasonal in communities, usually occurring as annual epidemics (2–5). Epidemiological data collected globally over the years confirm that the infection fades out regularly in communities for a few to many months during interepidemic periods (2, 4–7). However, the origin of seed viruses for new RSV epidemics remains unknown. This information might improve understanding of RSV transmission dynamics and aid in the design of future control and prevention strategies, including vaccines (8).

Seed viruses for new RSV epidemics may arise from two possible sources. Virus may continue to be transmitted at low levels during the interepidemic period but go undetected by commonly used hospital-based surveillance methods. It has been observed that some population groups, for example, HIV-infected individuals and infants, can shed virus for several weeks to months, with shedding extending into the period between epidemics (9, 10). Alternatively, local virus may disappear from the local population, creating an absolute requirement for new virus introduction from an outside population. Long-term detailed observations of the genetic relatedness of viruses circulating in communities over multiple successive epidemics may clarify the relative importance of each of these potential sources of virus for new RSV epidemics (11–13).

Of the several recognized viral causes of pneumonia, influenza type A viruses are the most studied in examining the origin of seed viruses for new epidemics and global virus ecology (14–17). Various mechanisms have been proposed, including the Southeast Asia epicenter model (15), the source-sink model (with the tropics serving as the source and temperate zones as the sink) (12, 14), and the global migrating viral metapopulation model (18). The clarity of understanding of influenza persistence has considerably improved with increased sampling in both epidemic and nonepidemic months and the incorporation of sequence analysis into epidemiological investigations (17, 19). Low-level circulation of influenza viruses during summer months and lineage regional persistence over multiple seasons are now recognized (17, 20, 21). However, minimal parallel work has been undertaken for the other viral causes of pneumonia, including RSV, to understand similarities and differences in patterns.

RSV isolates can be divided into two antigenic groups, A and B (22, 23), which are found to cocirculate in most epidemics but cycle in dominance over consecutive epidemics (24). Overall, RSV A is more prevalent (25). Within the groups, virus isolates display significant genetic variation most pronounced in the attachment (G) glycoprotein genomic region. Within RSV B, the focus of this report, 9 genotypes have been assigned based on phylogenetic analysis of the G gene: GB1 to -4, SAB1 to -4, and BA (26–29). It is apparent that some of the genotypes may have become extinct and that continuing genetic changes are accumulating in circulating ones. This occasionally gives rise to new genotypes (30–33). There is no overall consensus on the conditions to be fulfilled in order to designate a group of sequences as a new genotype (25, 34).

Multiple virus strains have been reported to cocirculate during local-community RSV epidemics (28, 35–37), and the dominant ones are replaced in subsequent epidemics, although not necessarily to the extent of total exclusion (27, 37–39). This pattern of strain dominance, evolution, and replacement at the local level suggests a role for strain-specific herd immunity, which favors the circulation of virus types heterologous to those that have recently circulated (25, 27, 37, 40). However, it is unknown to what extent these replacements could also be a reflection of the interepidemic bottleneck. Severe interepidemic bottlenecks, for example, reduce the chances of the previous epidemic virus types appearing again, as they may require reintroduction. Few studies have focused on this aspect of RSV molecular epidemiology with detailed phylogenetic analysis.

The current work examined the sequence relatedness of RSV B viruses that have been detected over 11 successive years in a rural coastal Kenyan community to identify virus sources for new epidemics. The viruses were detected through continuous surveillance of pediatric hospital admissions (2002 to 2012) and also in a community-based surveillance program (2002 to 2005). Over 600 partial G sequences of RSV B were phylogenetically analyzed and compared with contemporaneous data from elsewhere in Kenya and also data from 18 other countries (2002 to 2012). On the basis of pragmatic reasoning, we set out new definitions of genotypes and variants and criteria for assigning virus variants as locally persisting or newly introduced into the community during each epidemic. We find that observed RSV strains in each epidemic belong to numerous distinct variants that are seldom maintained in the local community from epidemic to epidemic. Comparison of viruses sampled over a wider scale, e.g., at the country level and globally, indicated both within- and between-country spread, though some variants are apparently confined geographically in their spread.

## MATERIALS AND METHODS

### Study location and population.

The study was conducted in Kilifi County of coastal Kenya, located north of Mombasa city (latitude of 3.6°S; area of about 12,464 km^2^). Kilifi County has a population of about 1 million and is predominantly rural, with a number of small townships. The area has a tropical climate with two rainy seasons (main rains in April to June and short rains in October to December). Subsistence farming and tourism are the primary economic activities in the region. International visitor arrivals are mostly but not exclusively from Western Europe (United Kingdom, Germany, Italy, and France) and the United States. The numbers of visitors from BRIC (Brazil, Russia, India, and China) countries, especially China and Brazil, are also reported to be on the rise (41). The specimens were collected during a birth cohort study following 635 children over a period spanning three consecutive epidemics between 2002 and 2005 (42) and a pediatric inpatient RSV surveillance study at the county's referral hospital, Kilifi County Hospital (KCH), between 2002 and 2012 (4). Details of the study designs, recruitment, and sampling procedures of the original studies have been described elsewhere (4, 42–44). All specimens (nasal washings, nasopharyngeal aspirates, or nasopharyngeal swabs) were obtained from children less than 5 years of age with signs of mild or severe acute respiratory infection or pneumonia.

### Laboratory procedures.

RSV was diagnosed in the clinical specimens by either an antigen or a nucleic acid detection test: indirect immunofluorescence assay (IFAT) (Light Diagnostics; Chemicon) and real-time reverse transcription PCR (RT-PCR), respectively (17, 18). For clinical specimens that were RSV positive by either method, viral RNA was extracted using a QIAamp RNA extraction kit (Qiagen Ltd., United Kingdom) from 140 μl of raw clinical specimen and used in part for molecular subtyping of the positive sampless into group A and B and also for partial G gene sequencing (44, 45). The RT-PCR procedure, sequencing procedure (based on the Sanger ABI dideoxy chain termination technology), and respective primers are as previously described (43). The analysis here focused on samples that were positive for RSV group B. The sequencing targeted the G gene ectodomain region from nucleotide 154 to the end relative to reference strain 8/60 (accession no. M73545). Contig assembly was undertaken in Sequencher v5.0.1 (Gene Codes Corporation).

### Comparison data set.

All RSV B G gene sequences deposited in GenBank as of 8 July 2015 whose sequenced regions overlapped the Kilifi sequences and derived from viruses collected between 2002 and 2012 were collated and phylogenetically compared with the Kilifi virus sequences. This analysis aimed to determine the relatedness of the Kilifi viruses to those circulating around Kenya and the rest of the world to understand their global context and to determine whether the patterns observed in this rural population are more widely extended. The data set comprised 833 sequences from 19 countries, including 116 sequences derived from parts of Kenya (Nyanza [*n* = 1], Nairobi [*n* = 7], and the Dadaab refugee camp, located near the Kenya-Somali border [(*n* = 108]). The other countries included South Africa, Argentina, Brazil, Cuba, Panama, Peru, the United States, Belgium, Germany, Italy, Netherlands, the United Kingdom, China, India, Pakistan, Saudi Arabia, South Korea, and Thailand. For computational tractability, the global comparison data were filtered to include only unique sequences for each country (or, for Kenya, for each region), a total of 629 sequences (for details, see Table S1 in the supplemental material).

### Sequence alignment and phylogenetic analysis.

Nucleotide sequences were initially aligned in the MAFFT v6.884b (46) program and then manually curated and trimmed to equal overlapping lengths in Se-Al software v2.0 (http://tree.bio.ed.ac.uk/software/seal/). To identify identical sequences over the region sequenced from the various sub-data sets (e.g., by country), an in-house Ruby script was used. Phylogenetic trees were generated using either maximum likelihood (ML) implemented in MEGA v5.2.1 or Bayesian evolutionary analysis and sampling of trees, as implemented in BEAST 1.8.2 software. The appropriate models of nucleotide sequence evolution for the various analysis data sets were identified in jModelTest v2.1.3 (47).

To identify the RSV B genotypes in Kilifi and global data sets, G sequences representative of previously identified RSV B genotypes (GB1 to GB4, SAB1 to SAB4, and BA) were included in the phylogenetic analysis. For ML trees, the robustness of the tree branching patterns was tested by bootstrapping with 1,000 iterations. The runs were conducted under the generalized time-reversible (GTR) model of evolution with gamma distribution and allowing for invariant sites. Sequences that clustered together with the known genotypes within the same branch with high bootstrap support (>70%) and/or within a defined genetic distance were considered to be of that genotype.

All BEAST runs assumed the HKY (Hasegawa, Kishino, and Yano) model of evolution with gamma distribution, invariant sites, and a demographic model of constant population size. Initial runs under the GTR model of evolution did not appear to converge. The analyses were set to 50 or 100 million steps with sampling after every 2,500 steps. The output was further analyzed only when the estimated sample size (ESS) for all parameters exceeded 200. Maximum clade credibility (MCC) trees were calculated using Tree Annotator 1.8.2. and visualized in FigTree v1.4.2 (http://tree.bio.ed.ac.uk/software/figtree/).

### Clustering analysis.

To verify relationships identified by phylogenetic tree analysis in terms of number of genotypes or clusters, the data sets were also analyzed using USEARCH clustering algorithm (48). This entailed first trimming the ends of the alignment to the longest shared sequence. To identify the cutoff that corresponded to genotype or a variant within a genotype, the identity threshold was varied in intervals of 0.005 step from 0.8 to 0.995, and the number of clusters observed was recorded. The analysis was redone with the carboxy-terminal G portion alone over the terminal 264 nucleotides (324 for genotype BA).

### New-variant introduction versus persistent variants.

We assumed a molecular-clock substitution rate model for RSV B in the G region we sequenced (ectodomain portion). From previous work, this has been estimated to be 1.95 × 10^−3^ (95% confidence interval [CI], 1.15 × 10^−3^ to 2.34 × 10^−3^) nucleotide substitutions per site per year (49). We calculated the maximum number of expected nucleotide differences between viruses collected in consecutive epidemics if persisting and locally evolving, using the upper limit of the substitution rate. When the nucleotide differences exceeded this expected number of changes, the virus variant was classified as newly introduced. Our approach is summarized in the following equation: *N_d_* = *L_a_* × *S_r_* × *T_e_*, where *N_d_* is the expected number of nucleotide differences, *L_a_* is the length of region analyzed, *S_r_* is substitution rate in the region analyzed, and *T_e_* is the time elapsed between the virus strains being determined as persisting or newly introduced. From this reasoning, we were able to assert that viruses with ≥4 nucleotide differences in comparison with viruses from preceding epidemics were newly introduced variants, while viruses with <4 nucleotide differences with viruses from the preceding 1 or 2 epidemics represented locally persisting variants. Variants assigned to either category were verified by examining their phylogenetic relatedness to other Kilifi sequences in the presence of the contemporaneous global background diversity data set (*n* = 629) originating from outside Kilifi.

### Epidemic designations and nomenclature.

RSV epidemic periods in Kilifi were defined using the hospital admissions data as previously described by Nokes et al. (4). The Kilifi epidemics are designated with the following format: year started (four digits)-year ended (two digits). RSV A or B group dominance was concluded if the group constituted ≥65% of total RSV-positive cases; otherwise, the groups were considered codominant. The definitions for the terms used in this report are provided in Table 1. The nomenclature of the sequences has the following format: country (KEN for Kenya)/unique identifier/month identified-year identified.

**TABLE 1:** Definition of terms^*a*^

Term	Definition
Unique sequences	The sequences within a set that possess at least one nucleotide difference among them in the region compared, i.e., the nonidentical sequences
Singleton	On a phylogenetic tree, a single sequence that is distant from other sequences due to possession of multiple nucleotide substitutions (≥4 nucleotide differences)
Genotype	Previously designated on the basis of phylogenetic analysis of the 2nd hypervariable region of G gene sequences; for RSV B, genotypes GB1 to -4, SAB1 to -4, and BA are included here; redefined here as showing >0.035 genetic distance from other sequences in the G gene ectodomain.
Variant	On a phylogenetic tree, a virus or group of viruses within a genotype that possesses ≥4 nucleotide differences in the G ectodomain region compared to other viruses and/or fall into a phylogenetic cluster away from the rest with >60% bootstrap statistical support
Newly introduced variant	A virus or a group of viruses whose sequence was not observed in previous epidemics from the same study population that (i) cluster together with a bootstrap support value of >60% or (ii) have <4 nucleotide differences among themselves and have ≥4 nucleotide differences from other viruses classified as distinct variants
Persistent variant	A virus (or a group of viruses) whose sequence(s) has <4 nucleotide differences in the G region from a variant detected in the immediate 1 or 2 preceding epidemics

a Definitions relevant to the G ectodomain region of the RSV genome we sequenced.

### Ethics statement.

The samples obtained in Kilifi were collected after informed written consent was obtained from each child's guardian or parent. The study protocols were approved by the Kenya Medical Research Institute (KEMRI) Ethical Review Board, Kenya, and the Coventry Research Ethics Committee of the United Kingdom (4, 42).

### Nucleotide sequence accession numbers.

All Kilifi sequences reported for the first time have been deposited in GenBank under the accession numbers KP862056 to KP862529.

## RESULTS

### Seasonality and genotyping of RSV B viruses in Kilifi.

**FIG 1:**
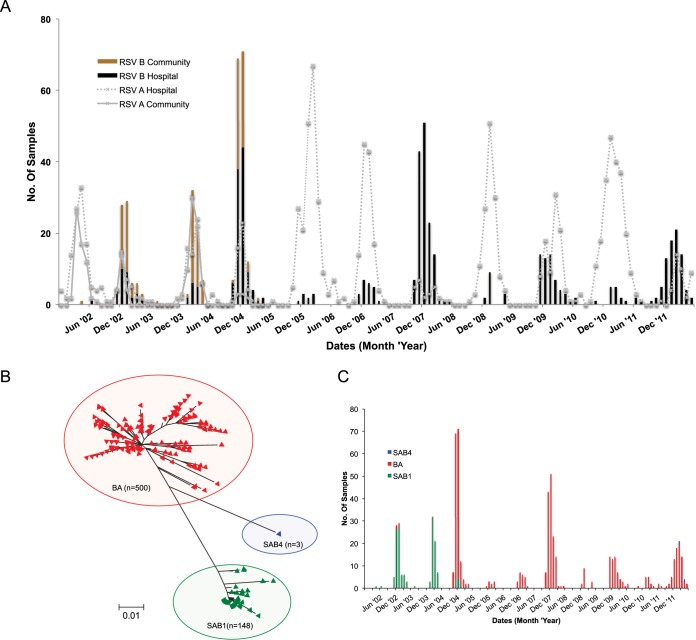
Temporal patterns and genetic diversity in RSV strains from Kilifi 2002-12. (A) Combined monthly detection frequency of RSV A and B viruses from both the KCH child inpatient surveillance study, 2002-12, and the community birth cohort study, 2002-05. The bars show RSV B detection (black, hospital study; brown, cohort study). The gray show RSV A detection (dotted line, hospital study; solid line, cohort study). (B) ML phylogenetic tree showing the relationship of all 651 sequences. The Kilifi sequences formed three major clusters: SAB1 (green), BA (red), and SAB4 (blue). (C) Monthly occurrence of the 3 genotypes from January 2002 to June 2012.

Between 1 January 2002 and 31 June 2012, 11 RSV epidemic peaks occurred in Kilifi, as observed from the pediatric inpatients who were positive at admission (2002-12) and the birth cohort follow-up (2002-05) (Fig. 1A). The epidemics occurred on an annual basis, mostly from November of one year to between June and August of the following year, with the peak falling outside the rainy season. The monthly prevalence of RSV cases at the hospital mirrored the observations in the community-based surveillance (2002-05), with clear interepidemic periods seen in both populations. Both RSV A and RSV B were detected in all the epidemics, but their numbers fluctuated from epidemic to epidemic; RSV B was dominant in two of the epidemics (2004-05 and 2007-08) and codominant with RSV A in 4 of the epidemics (2002-03, 2003-04, 2009-10, and 2011-12) (Fig. 1A). Overall, RSV A was more prevalent. The hospital surveillance identified a total of 574 RSV B infections; 488 (85.0%) of the viruses from these infections were sequenced in the G ectodomain region, while the birth cohort study detected 163 RSV B infections, viruses from all of which were G sequenced.

Alignment and phylogenetic analysis of the two data sets combined demonstrated that the Kilifi sequences formed three major clusters on the ML phylogenetic tree (Fig. 1B). When compared to the genotype reference sequences, the clusters corresponded to genotype BA (500 [76.8%]), which carries the 60-nucleotide duplication in the carboxy terminus G region, to genotype SAB1 (148 [22.7%]), and to genotype SAB4 (3 [0.5%]).

The number of cases for each of the three genotypes we detected by month and by epidemic for the entire period is shown in Fig. 1C and in Table S2 in the supplemental material. Genotype SAB1 was the most prevalent group B genotype during the first three epidemics in the surveillance, although only 2 cases were detected in the first epidemic, which was otherwise dominated by RSV A viruses (Fig. 1A). The BA genotype was first observed in 2002-03, was dominant in the 2004-05 epidemic, and was the only RSV B genotype detected thereafter until the 2011-12 epidemic, when 3 SAB4 viruses were observed for the first time in the Kilifi community (Fig. 1C).

### Within- and between-genotype diversity.

The G gene region sequenced for the 651 Kilifi viruses varied in length between 603 and 678 nucleotides due to the presence of a 15-nucleotide (an SAB1 strain) or a 60-nucleotide (genotype BA viruses) duplication and a 6-nucleotide deletion in some. Of the 651 sequences, 369 (56.7%) shared an identical sequence with at least one other sample, while 282 (43.3%) viruses gave unique sequences, i.e., they differed by at least one nucleotide. Within genotype SAB1, there were 57 (38.5%) unique sequences; within genotype BA, there were 221 (44.2%) unique sequences, while all genotype SAB4 sequences were identical. The intergenotype genetic distances of unique sequences for SAB1 versus BA, SAB1 versus SAB4, and BA versus SAB4 were 0.072, 0.079, and 0.063, respectively. The mean genetic distances of the unique sequences within SAB1 and BA genotypes were 0.007 and 0.019, respectively, corresponding to ∼4 and 13 nucleotide differences.

**FIG 2:**
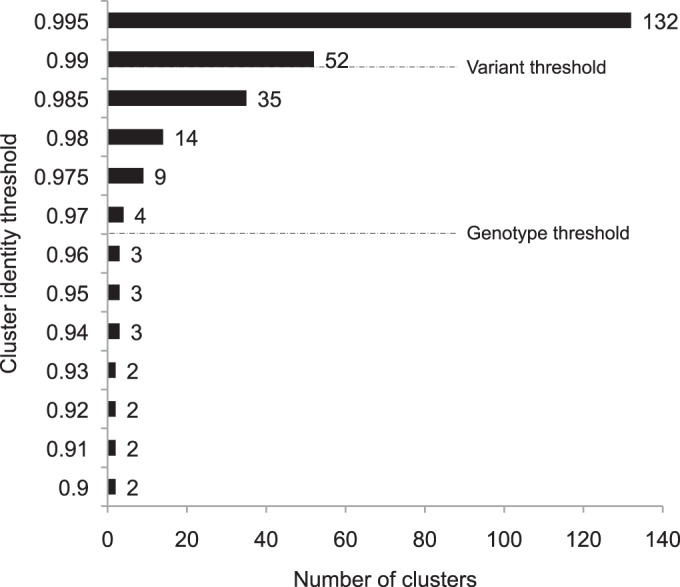
Clustering of the group B viruses identified in Kilifi (2002-12) to define genotype and variant. (C) The ectodomain region of Kilifi viruses was analyzed with the USEARCH algorithm. Thresholds corresponding to genotype (0.965) and variant (0.99), i.e., corresponding to the 4-nucleotide definition (see Materials and Methods) are indicated.

The criteria for assigning RSV isolates to genotypes, subgenotypes, or variants have not been consistent across laboratories. Different genes, analytical methods, and levels of diversity have been considered to define genotypes (25, 34). However, phylogenetic analysis of G gene sequence data, in particular, the second hypervariable region, has been the most used. Based on number of nucleotide differences, in Fig. 2 we show the number of different clusters identifiable (that would correspond to genotypes or variants) for the Kilifi viruses as we increased the identity threshold from 0.8 to 0.995 in the USEARCH algorithm. Relaxing the cluster diversity threshold (raising the value toward its maximum) from around 0.96 resulted in a rapid nonlinear increase in the number of clusters. The genotype identity threshold of 0.965 gave us the three genotypes identified in Fig. 2, corresponding to a genetic distance of 0.035 over the 678-nucleotide region we sequenced. When the carboxy-terminal portion alone was evaluated, this corresponded to a genetic distance of 0.065. Thus, different thresholds will be applicable depending on the size and evolutionary nature of the genomic fragment analyzed.

### Within- and between-epidemic genetic diversity.

**FIG 3:**
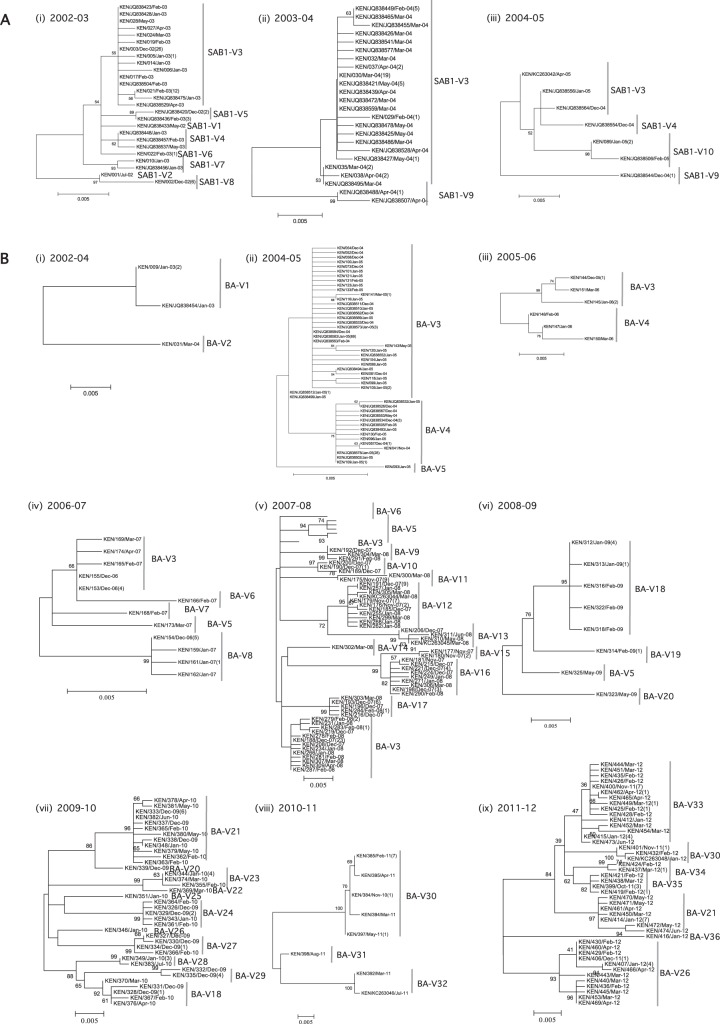
ML phylogenetic trees of G sequences from the individual Kilifi epidemics. (A) Genotype SAB1 viruses detected in 4 epidemics (2001-02 viruses were combined with 2002-03 sequences in panel i). (B) Genotype BA viruses detected in 10 epidemics (epidemic 2002-03 and epidemic 2003-04 viruses were combined in panel i). The trees include only unique sequences for each epidemic. Variant names are next to the phylogenetic clusters, e.g., SAB1-V1 for SAB1 variant 1. A repeated variant name implies persistence between epidemics. Numbers in parentheses are numbers of other sequences that were identical to the sequence shown.

For each of the genotypes we detected, we examined its genetic variability in each epidemic in which it occurred. The analysis was possible only for genotype SAB1 and genotype BA, as there were too few sequences for genotype SAB4. For both SAB1 and BA, much diversity was observed in viruses cocirculating in the epidemics (Fig. 3). Based on our criteria defined in Table 1, we assigned the viruses falling into different phylogenetic clusters to distinct variants to allow further analysis. The criterion for designation of a variant was at least 4 nucleotide differences with any other virus (Fig. 2). A total of 47 distinct variants were assigned from 651 sequences (Fig. 3; also, see Table S3 in the supplemental material). Genotype SAB1 viruses were detected over the period from 2002 to 2005, with a total of 148 viruses constituting 10 distinct variants. Genotype BA viruses were detected from 2003 through 2012, with a total of 500 viruses representing 36 distinct variants. The number of variants observed varied by epidemic (median, 5; mean, 5.8; range, 2 to 12) (see Table S2 in the supplemental material). Some of the variants in the epidemics were detected as singletons, i.e., represented by single viruses.

**FIG 4:**
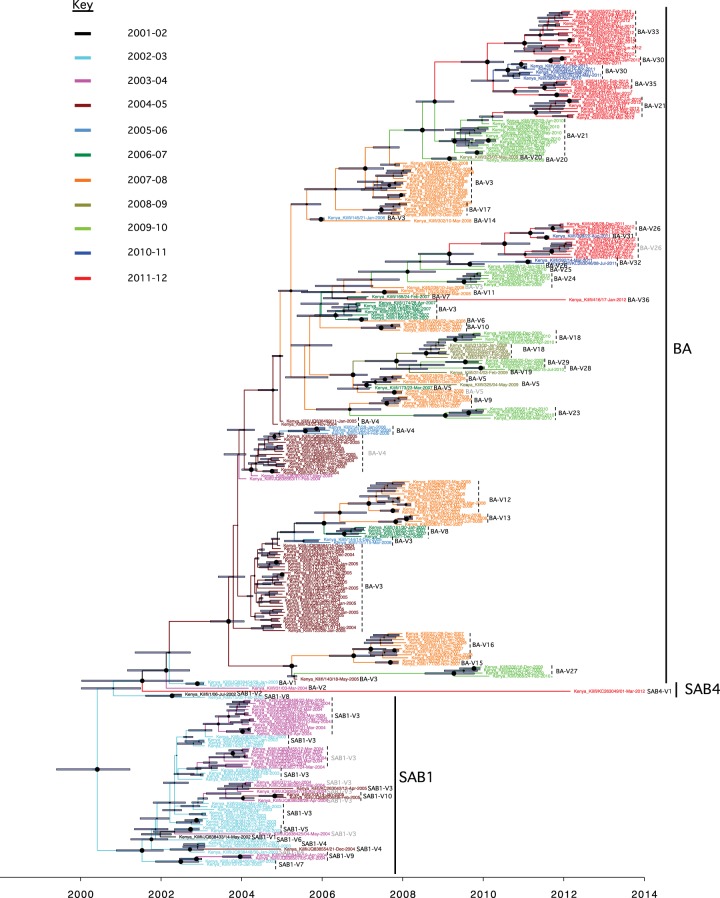
BEAST tree showing the phylogenetic and temporal placement of the 282 unique Kilifi sequences reported in this study. The taxon names are colored by epidemic: black, 2001-02; cyan, 2002-03; pink, 2003-04; maroon, 2004-05; light blue, 2005-06; green, 2006-07; orange, 2007-08; dark green, 2008-09; bright green, 2009-10; blue, 2010-11; red, 2011-12. Variant names that were derived in Fig. 3 are indicated next to the sequence clusters or singletons. The node bars indicate the 95% highest posterior density (HPD) height interval, while the sizes of the filled circles on the nodes indicate the level of posterior support for the associated branch. Variant names in gray indicate those observed on multiple branches during the same epidemic.

The cocirculation of multiple group B variants during the individual Kilifi epidemics was also identifiable on phylogenetic analysis of the combined unique sequences from all the epidemics (2002-12). A molecular-clock phylogeny including all the Kilifi RSV B viruses identified over the 11 epidemics is shown in Fig. 4. Taxa of viruses from the same epidemic are identified by same color. An equivalent phylogenetic tree generated by the ML method is given in Fig. S4 in the supplemental material. Overall, viruses from the same epidemic did not form monophyletic groupings; instead, multiple phylogenetic clusters were observed, confirming cocirculation of multiple variants in a single epidemic. For instance, the 2007-08 epidemic recorded the highest number of cocirculating variants (Fig. 4, taxon names shown in orange; a total of 12 variants), some of which were interspersed with variants from other epidemics. A few phylogenetic clusters (that corresponded to variants we assigned) contained sequences from multiple epidemics, indicating variant persistence (or repeated reintroduction) over multiple epidemics, e.g., genotype BA-V3 (outlined with a gray shaded rectangular box in Fig. S4 in the supplemental material), which was observed in 2004-08.

### Variant persistence patterns in Kilifi (2002-12).

Most of the variants were detected in only a single epidemic (see Fig. S5 in the supplemental material). Among the 10 genotype SAB1 variants, seven (70.0%) were each observed in only a single epidemic and were undetectable thereafter, while the remaining three were observed in multiple epidemics: one variant in three consecutive epidemics (variant SAB1-V3) and the other two in two epidemics, of which one was in consecutive epidemics (SAB1-V9) and the other was in nonconsecutive epidemics (SAB1-V4) (see Fig. S5A in the supplemental material). Among the 36 genotype BA variants, 28 (77.7%) were detected in only a single epidemic. The remaining 8 variants were detected over multiple epidemics: 6 in two epidemics (16.7%) (two of which arose in nonconsecutive epidemics, BA-V21 and BA-V26), one in four consecutive epidemics (2.7%) (BA-V3), and another one in four nonconsecutive epidemics (2.7%) (V5) (see Fig. S5B in the supplemental material). For the BA variants that occurred in only one epidemic, 4 were observed for the first time in the last epidemic of the surveillance, and thus it is unknown from the current analysis if they reappeared in the subsequent new epidemics in the county.

Thus, overall, we estimated at least 47 introductions (including one for SAB4) (Fig. 4) of novel group B variants into the Kilifi district over the study period (see Table S3 in the supplemental material), translating to an average of 4 new variant introductions per epidemic (range, 0 to 9). Importantly, the number of introductions could be higher, as some variants showed some intradiversity, which may reflect multiple independent introductions of closely related strains (Fig. 4; also, see Fig. S4 in the supplemental material).

### Incremental evolutionary relatedness of Kilifi variants through time.

**FIG 5:**
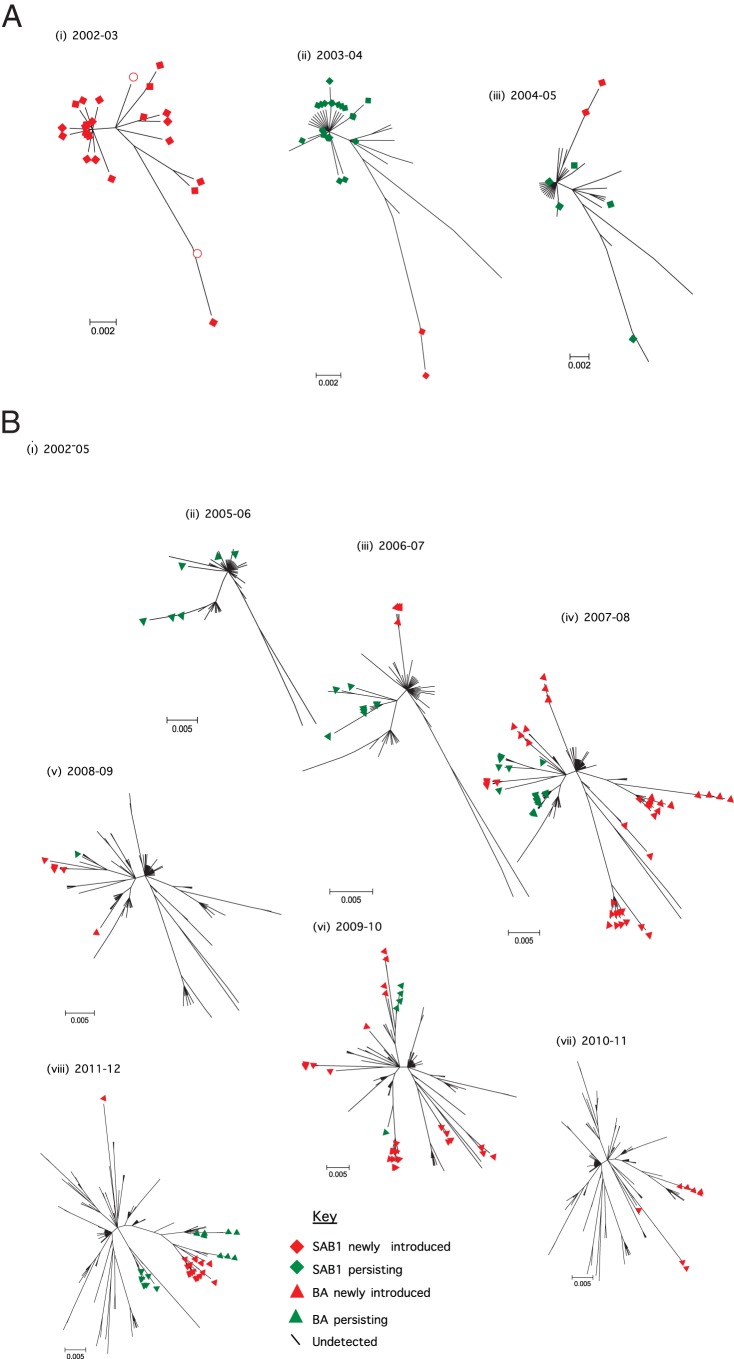
Radial ML phylogenetic trees showing the incremental genetic relatedness of each new epidemic strains sequences relative to the previous epidemic sequences from Kilifi. (A) Genotype SAB1 viruses detected in 4 epidemics (2001-02) viruses (open red circles) and 2002-03 epidemic viruses are combined in panel i. (B) Genotype BA viruses detected in 10 epidemics (2002-03, 2002-04, and 2004-05 viruses are combined in panel i). Throughout the trees, the strains that were perceived to be of newly introduced variants are represented by a red symbol; those perceived to belong to variants that persisted from previous Kilifi epidemics are represented by a green symbol. Tips without a marker imply that the associated strains had been observed in previous epidemics but were not observed in the current epidemic.

For every new epidemic, we evaluated the G evolutionary relationship of the circulating viruses to those from the preceding epidemics. This allowed an understanding of the genetic relatedness of these viruses year by year and the identification of apparently “persisting variants” and “newly introduced variants” in the Kilifi community. Figure 5A shows the incremental evolutionary relationships of the genotype SAB1 viruses identified with each new epidemic that was observed from 2002 to 2005. Figure 5B shows the incremental evolutionary relationship of the genotype BA viruses detected with each new epidemic from 2003 to 2012. Over time, the complexity of the radial trees increased for both genotypes as the sample depth increased. New branches frequently emerged, and there was accumulation of diversity with each new epidemic. This was especially evident in genotype BA, which was observed over the longest timespan (Fig. 5B). Visually, it appears that length of tree growth, i.e., the extent of genetic divergence from year to year, diminishes beyond epidemic 2007-08 for the BA genotype, as though some constraint on the rate of genetic diversification had set in.

### Infection prevalence: introduced versus persistent variants.

We evaluated the contribution of newly introduced variants versus persisting variants in the total infections observed in each of the 11 epidemics (see Fig. S6 in the supplemental material). Overall, of the 651 viruses we sequenced, 473 (72.7%) belonged to the “newly introduced variants” category while 178 (27.3%) belonged to the “persisting variants” category after initial introduction in previous epidemics. The relative contribution of these two sources in the individual epidemics varied (see Fig. S6A in the supplemental material). The 2005-06 epidemic did not record any new RSV B variant introduction (epidemic RSV A dominated) (Fig. 1), while the 2002-03 and 2010-11 epidemics did not record any persisting group B variant infections. The relationship between the number of variants introduced or persisting did not seem linear to the number of group B infections detected in epidemics (see Fig. S6B in the supplemental material).

### Relatedness of Kilifi and global RSVB viruses (2002-12).

**FIG 6:**
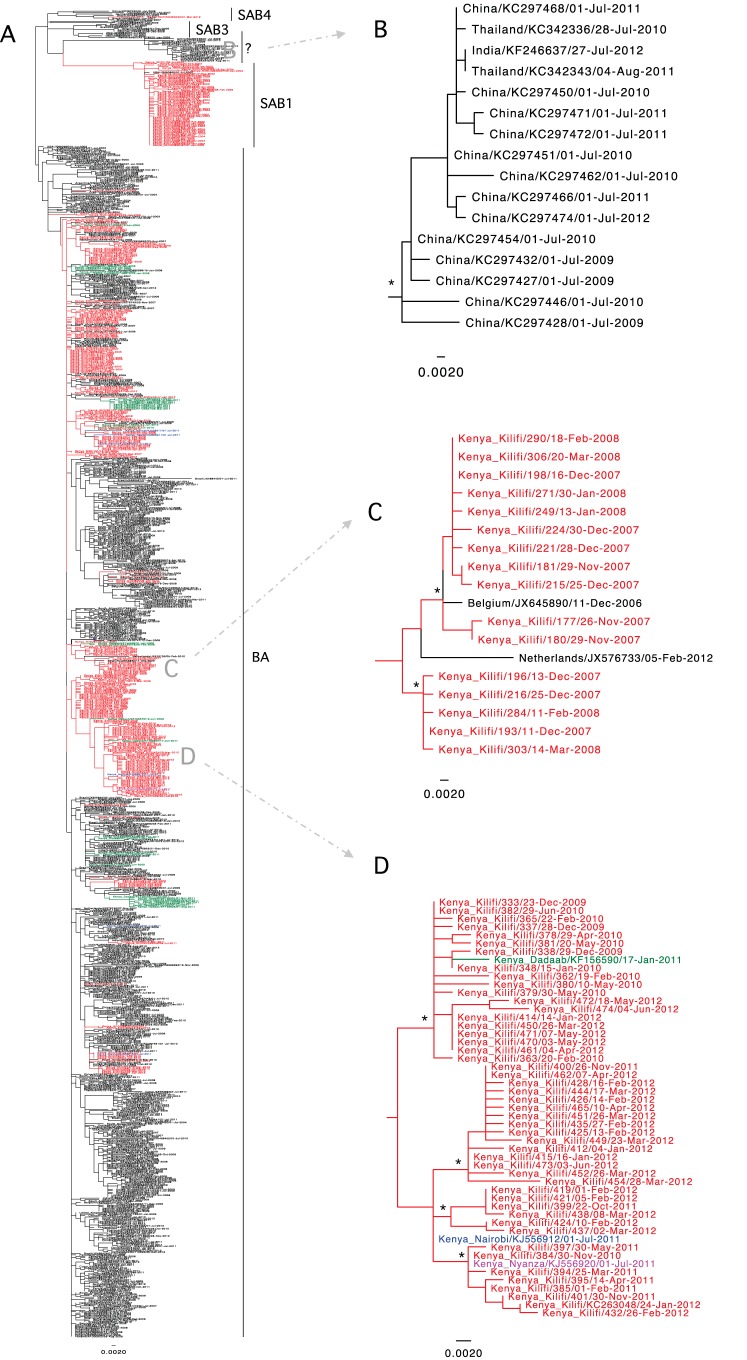
Global context of the Kilifi group B viruses. (A) ML phylogenetic tree comparing Kilifi 2002-12 unique sequences (*n* = 282) and contemporaneous data from around Kenya and 18 other countries (*n* = 629). The taxon names of Kenyan sequences are in red for Kilifi, blue for Nairobi, green for Dadaab, and pink for Nyanza. Taxon names of sequences from outside Kenya (background diversity) are in black. (B) Example of a cluster without a Kenyan sequence. (C) Example of a cluster with a majority of Kenyan sequences but with a few sequences from abroad, as well as supporting global migration of the virus. (D) An example of a cluster of Kilifi sequences intermixing with sequences from other parts of Kenya to show case-within-country virus migration and perhaps epidemic seeding.

The G sequences of Kilifi viruses were compared with those of viruses identified during the same period from around Kenya and 18 other countries. The ML phylogenetic clustering of these sequences (*n* = 911) is shown in Fig. 6. An equivalent molecular-clock-calibrated phylogeny is given in Fig. S7 in the supplemental material. Taxon names of sequences from around Kenya are shown in color (Kilifi, red; Dadaab refugee camp, green; Nairobi, blue; Nyanza, pink). Sequences from outside Kenya are shown in black. Sequences originating from Kilifi and/or other parts of Kenya did not form a single monophyletic group; instead, they were interspersed mostly as multiple small to medium-sized clusters located separately or with clusters of viruses from other countries (Fig. 6A).

In Fig. 6B to D, we zoomed in to understand the composition of the observed clusters, especially those with relatively high bootstrap support (>55%). Various types of cluster composition were seen: (i) clusters of virus variants circulating abroad but never sampled in Kilifi (Fig. 6B), (ii) clusters of virus variants predominantly observed in Kilifi but with minor representation of viruses sampled outside Kenya (e.g., the United States and Europe [for example, Belgium and Netherlands]) (Fig. 6C), perhaps depicting origins or subsequent export to these places, and (iii) clusters of virus variants found principally in Kilifi, with a minor representation found elsewhere in Kenya (Fig. 6C). These observations were consistent with both within-country and global migration in RSV maintenance in its human populations.

In our final analyzed global data set, a total of four known genotypes were identified that circulated during the encompassed period: genotype SAB1 (*n* = 58), genotype SAB3 (*n* = 12), genotype BA (*n* = 818), and genotype SAB4 (*n* = 7) (Fig. 6A). The remaining sequences (*n* = 16) formed a unique cluster outside the known genotypes. This aberrant group of sequences originated mainly from China, with two collected in Thailand and one from India (Fig. 6B). Genotype BA was the most abundant during this study period in all countries included in our analysis.

## DISCUSSION

The occurrence of discrete regular RSV disease epidemics in communities is a well-known phenomenon (5, 50, 51). However, unlike with influenza viruses, little attention has been paid to understanding the source of RSV seed viruses for new epidemics, patterns of introduction, and persistence of variants in local communities and how these interact to produce RSV global epidemiology. For influenza virus, studies suggest that a number of factors come into play, including climate, host, and virus factors (17, 52). Influenza type A virus in particular and RSV have important epidemiological similarities (e.g., seasonal transmission, antigenic variation in surface proteins, and transmission via respiratory secretions) but also have key differences; e.g., influenza A virus exhibits linear strain replacement and extinction, a segmented genome (with frequent reassortment), and a zoonotic reservoir, which are absent in RSV.

Here we present a detailed longitudinal phylogenetic study of RSV group B viruses detected at the Kenyan coast over 11 consecutive epidemics. The study examined relatedness of RSV strains within and between epidemics in this tropical rural location, comparing them with those occurring in other parts of Kenya and globally. Our findings suggest that seed viruses for new RSV epidemics in this community were primarily new-variant introductions, although with occasional variant persistence. Throughout the surveillance, epidemic peaks comprised multiple cocirculating group B variants, with an average of 4 variants per epidemic, the vast majority of which occurred only in one epidemic. No variant was detectable for more than four consecutive epidemics. Up to 9 variant introductions in a single epidemic were recorded with RSV group B alone, and this demonstrates that virus entry into this community is repeated through a single or multiple entry points.

The sources of introduced variants are likely to be numerous. Introduction from elsewhere in Kenya is likely to be due to seasonal migration, e.g., for holidaying at the coast, which is a popular destination. The viruses observed in other parts of Kenya (Nairobi, Nyanza, and Dadaab refugee camp, despite the smaller sample size) were often sandwiched in clusters comprising primarily Kilifi viruses, supporting the notion that within-country, between-community transmission or epidemic seeding is likely to be key in RSV persistence. In addition, Kilifi lies on a main road from the major port of Mombasa toward the northeast of Africa and is also a popular tourist destination for visitors from Western Europe and the Americas. Thus, it is no surprise that our comparison of the Kilifi viruses with contemporaneous data from 18 countries revealed close genetic relatedness of viruses from Kilifi with those from Belgium, Netherlands, Brazil, Cuba, the United States, and occasionally India and South Africa (perhaps reflecting introductions via international tourist visits). Importantly, there were also variants observed in viruses from a number of other countries, including countries in Western Europe, Thailand, Cuba, Panama, and South Africa, that were not detected in Kilifi throughout our surveillance period.

Within Kilifi, a smaller proportion of variants were observed in multiple epidemics up to four consecutive epidemics (i.e., 5 years maximum). This finding suggests that a limited number of variants might survive in the community through the interepidemic troughs. Alternatively, it is possible that these variants are reintroductions of strains that had previously circulated in the community and might have migrated to a nearby community, such as the city of Mombasa (data were not available for inclusion in this analysis). Such persistence of variants may be a stochastic phenomenon in the maintenance of chains of transmission or may be due to a variant's intrinsic capability to evade the population immune responses.

Our findings here of (i) cocirculation of numerous RSV genetic variants during individual RSV epidemics, (ii) different genotypes/variants predominating in consecutive epidemics, and (iii) some previously detected genotypes/variants being absent in subsequent RSV epidemics in the Kilifi community have also been observed in other long-term RSV molecular epidemiology studies. Examples include the study by Cane et al. in Birmingham, United Kingdom (13, 37), and that by Peret et al. in Rochester, USA (27). Our study reinforces these observations with detailed phylogenetic comparisons of viruses from a tropical developing country and an expansive comparison with contemporaneous viruses derived globally. Further, it has been observed that even with influenza virus, multiple virus introductions into a community occur to make up an epidemic for both seasonal flu (11) and pandemic flu, e.g., as observed with 2009 H1N1 in the United Kingdom (53). This striking similarity of RSV and influenza virus with regard to community epidemic seeding could actually be a universal phenomenon for seasonally recurring respiratory viruses.

We suggest explicit criteria for assigning locally sequenced RSV strains into genotypes and variants. A genetic distance of 0.035 from other viruses (over the ectodomain region) is recommended to assign a genotype. This is based on the clustering patterns observed in the USEARCH algorithm and intergenotypic distances of the known genotypes and observed RSV rates of nucleotide fixation. However, the occurrence and spread of abrupt large deletions or insertions, as in the case of genotypes BA and ON1, will be another situation leading to direct designation of a new genotype. Our attempt to clarify this is motivated by the absence of a systematic RSV genotyping system based on genetic similarity (34). The assignment of sequences within genotypes into variants in this study helped us define an epidemiologically functional unit to better understand the dynamics of strain introduction and persistence in our local population. The cutoff (≥4 nucleotide differences), however, can be adjusted to fit the question being addressed.

Our study is not without limitations. First, it is likely that there were some variants that circulated in a number of epidemics in this community that were not sampled during the surveillance and thus were recorded as new introductions or disappeared variants, as our sampling targeted only individuals with acute respiratory infection (ARI) symptoms (54). A future study should include sampling across all age groups and diagnosis of asymptomatic individuals to identify any silent variant circulation. Second, our analysis here examined relatedness of the viruses only in the G ectodomain region. Future studies to extend this analysis will give a finer resolution if whole-genome sequences are examined to provide greater statistical support to perceived persistence or new-variant introductions and also to our recent observation that viruses can be identical with regard to G but possess several nucleotide differences elsewhere in their genomes (55). Third, assertions of origins of the variant introductions are unavoidably tentative due to the very limited number of archived RSV sequences from around Kenya and East Africa and, unlike for influenza A virus, the absence of a comprehensive worldwide RSV surveillance network. We also note that most RSV molecular epidemiology studies around the globe to date report sequences of only the second hypervariable region of the G ectodomain alone, which is adequate for genotyping but not appropriate in studies aiming to understand origins due to lower resolution of distinct variants.

In conclusion, our analysis of long-term sequence data from Kilifi shows that at the local level, RSV may not be endemic in the sense that persistence between recurrent epidemics is primarily sustained by frequent reseeding into the community from both close and distant locations to facilitate new seasonal epidemics. The data suggest that introduced variants may be predominantly from elsewhere in Kenya or the immediate region rather than from the global pool. A small proportion of variants appeared to survive locally for a few years but without causing the majority of new epidemic infections or large epidemics. This level of detail of analysis of circulating RSV has not been undertaken previously for a single location in a longitudinal manner. For a further and more precise understanding of the location source of RSV variants identified in Kilifi and the spread pathways and networks, sampling throughout Kenya with comprehensive sequencing (full G or WG) and comparison with representative sequence data sets sampled regionally (e.g., across East and Central Africa) and globally are suggested.

## Supplementary Material

Supplemental material
