# Supplementary material for: Successive Respiratory Syncytial Virus Epidemics in Local Populations Arise from Multiple Variant Introductions, Providing Insights into Virus Persistence
Source: J Virol. 2015 Sep 9;89(22):11630–42. doi: 10.1128/JVI.01972-15 (PMC4645665; doi:10.1128/JVI.01972-15)
Supplement: Supplemental material [file supp_89_22_11630__index.html]

Supplemental material 

# Successive Respiratory Syncytial Virus Epidemics in Local Populations Arise from Multiple Variant Introductions, Providing Insights into Virus Persistence

## Supplemental material

- Supplemental file 1 -

  Table S1 (Details of the sequences included in the global-comparison dataset.)

  Table S2 (Number of RSV-B-sequenced specimens by epidemic and genotype in Kilifi, 2002–2012.)

  Table S3 (Number of group B variants by epidemic and genotype introduced and persisting over 11 epidemics in Kilifi.)

  Fig. S4 (ML phylogenetic tree of the 282 unique sequences reported in this study.)

  Fig. S5 (Temporal epidemic map showing the occurrence patterns of the variants that were detected in the Kilifi surveillance, 2002– 2012.)

  Fig. S6 (Infection prevalence in relation to number of introduced versus persistent variants.)

  Fig. S7 (BEAST tree showing the phylogenetic and temporal placement of the 911 unique Kilifi sequences plus global sequences.)

  PDF, 502K
