# Supplementary material for: Successive Respiratory Syncytial Virus Epidemics in Local Populations Arise from Multiple Variant Introductions, Providing Insights into Virus Persistence
Source: J Virol. 2015 Sep 9;89(22):11630–42. doi: 10.1128/JVI.01972-15 (PMC4645665; doi:10.1128/JVI.01972-15)
Supplement: Supplemental material [file JVI.01972-15_zjv999090977so1.pdf]

Successive Respiratory Syncytial Virus Epidemics in Local Populations Arise from Multiple Variant Introductions Providing Insights into Virus Persistence

Charles N. Agoti[1, 2, #], James R. Otieno[1], Mwanajuma Ngama[1], Alexander G. Mwihuri[1], Graham F. Medley[3], Patricia A. Cane[4] and D. James Nokes[1, 5]

Authors' affiliations

1. *Department of Epidemiology and Demography, Kenya Medical Research Institute (KEMRI) – Wellcome Trust Research Programme, Kilifi, Kenya*
2. *Department of Biomedical Sciences, Pwani University, Kilifi, Kenya*
3. *Department of Global Health & Development, London School of Hygiene and Tropical Medicine, London, UK*
4. *Public Health England, Salisbury, UK*
5. *School of Life Sciences and WIDER, University of Warwick, Coventry, UK*

#Corresponding author

Email: cnyaigoti@kemri-wellcome.org (CNA)

Country	Total Sequences	Unique	Period	Genotype (s)
Argentina	39	29	2002-2008	BA
Belgium	109	97	2006-2011	BA, SAB3
Brazil	103	73	2002-2012	BA, SAB3, SAB4
China	73	65	2007-2012	BA, Untyped
Cuba	40	36	2009-2011	BA
Germany	3	3	2008-2009	BA, SAB4
India	35	30	2008-2012	BA, Untyped
Italy	10	8	2009	BA
Kenya: Kilifi	651	282	2002-2012	BA, SAB1, SAB4
Kenya: Dadaab	108	33	2007-2011	BA
Kenya: Nairobi	7	6	2011	BA
Kenya: Nyanza	1	1	2011	BA
Netherlands	48	44	2002-2012	BA
Pakistan	2	2	2012	BA
Panama	21	14	2008-2012	BA
Peru	34	19	2007-2012	BA
Saudi Arabia	2	2	2008-2009	BA
South Africa	98	77	2006-2011	BA
South Korea	24	19	2008-2010	BA
Thailand	30	30	2006-2011	BA, Untyped
UK	20	19	2005-2010	BA
USA	26	22	2002-2012	BA, SAB1, SAB3
**Total**	**1484**	**911**	**-**	**-**

S1 Table. Details of the sequences included in the global comparison dataset

S2 Table. Number of RSV B sequenced specimens, by epidemic and genotype in Kilifi, 2002-12

Peak epidemic month	**Total No. Of Sequences**	**No. Sequences Unique**	**No. Of Group B Viruses By Genotype[1]**			**No. Of. Variants By Genotype[2]**		
			SAB1	BA	SAB4	SAB1	BA	SAB4
2001-02	2	2	2	0	0	2	-	0
2002-03	78	25	74	4	0	6	1	0
2003-04	63	25	62	1	0	2	1	0
2004-05	167	55	10	157	0	4	3	0
2005-06	9	6	0	9	0	-	2	0
2006-07	22	12	0	22	0	-	5	0
2007-08	141	61	0	141	0	-	12	0
2008-09	14	8	0	14	0	-	4	0
2009-10	58	37	0	58	0	-	11	0
2010-11	17	8	0	17	0	-	3	0
2011-12	80	46	0	77	3	-	7	1
Total	651	285	148	500	3	14	49	1

[1]Only RSV Genotype SAB1, BA and SAB4 were detected in the Kilifi dataset

[2]Some Variants are counted in multiple epidemics as they persisted or recurrent

S3 Table. The number of group B variants introduced and persisting, by epidemic and genotype, over 11 epidemics in Kilifi

Epidemic	**Introductions**				**Persistence**			
	SAB1	BA	SAB4	Total	SAB1	BA	SAB4	Total
2001-02	2	0	0	2	-	0	0	-
2002-03	6	2	0	8	0	0	-	0
2003-04	1	1	0	2	1	0	-	1
2004-05	1	3	0	4	3	0	-	3
2005-06	0	0	0	0	0	2	-	2
2006-07	0	3	0	3	0	2	-	2
2007-08	0	9	0	9	0	3	-	3
2008-09	0	3	0	3	0	1	-	1
2009-10	0	9	0	9	0	2	-	2
2010-11	0	3	0	3	0	0	-	0
2011-12	0	4	1	5	0	3	-	3
Total	10	36	1	47	4	13	-	17

[1]Strains were considered as persisting if they possessed ≤4 nucleotide changes from strains that had been observed in the previous epidemic (Table 1).

[2]There was no preceding epidemic, hence it was not possible to distinguish persistent from newly introduced variants.

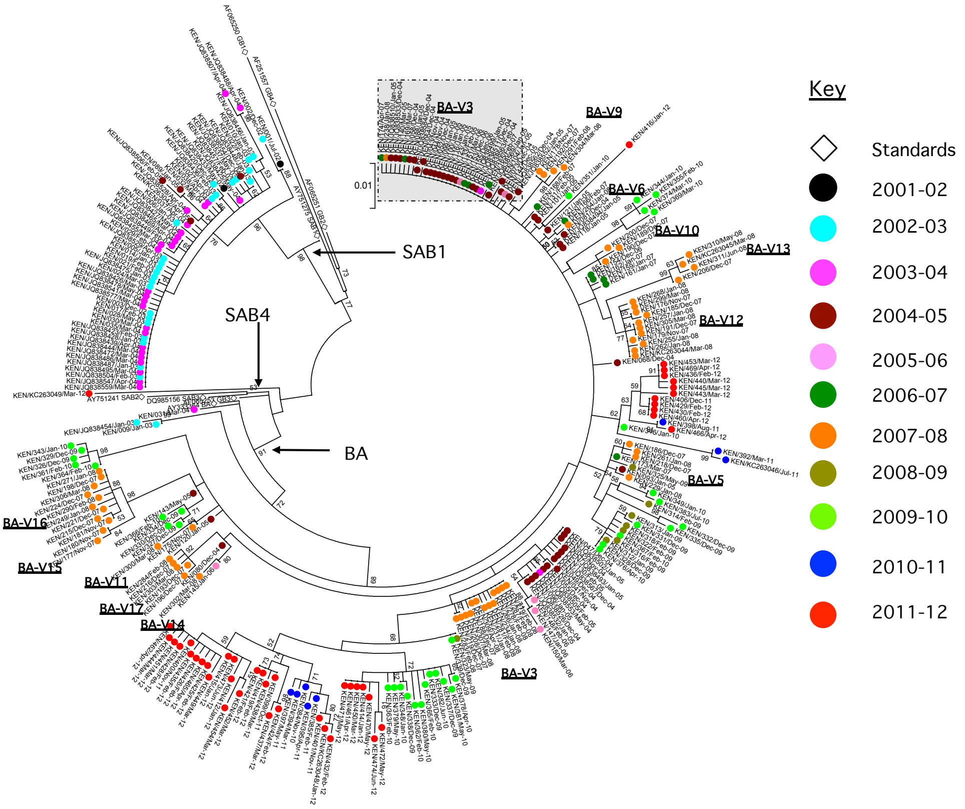

S4 Fig. A ML phylogenetic tree of the 282 unique sequences reported in this study. The filled circles preceding the taxon names are colored by epidemic: black – Early 2002, cyan - 2002-03, pink – early 2004, maroon – 2004-05, salmon pink – 2005-06, green – 2006-07, orange – 2007-08, dirty green – 2008-09, bright green – 2009-10, blue – 2010-11 and red - 2011-12. Eight additional sequences representative, of known genotypes are are included as standards. These are predeed by an empty black circle. As an example, variants correspounding to the 2007-08 epidemic (Fig. 3) have been indicated next to the sequences.

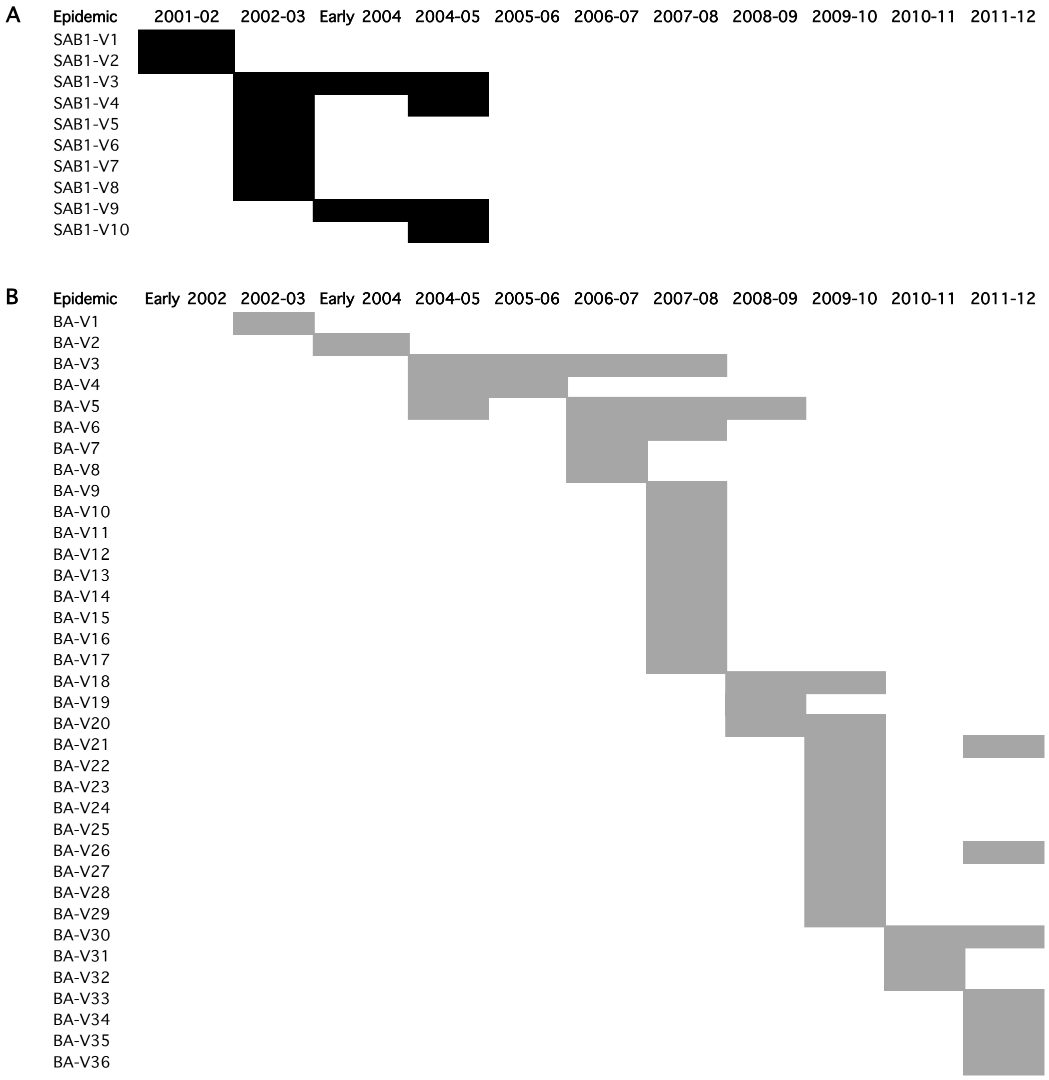

S5 Fig. A temporal epidemic map showing the occurrence patterns of the variants that were detected in the Kilifi surveillance, 2002-2012. **Panel A.** Genotype SAB1. **Panel B.** Genotype BA. The shaded boxes refer to the specific epidemics within which these variants were detected.

A

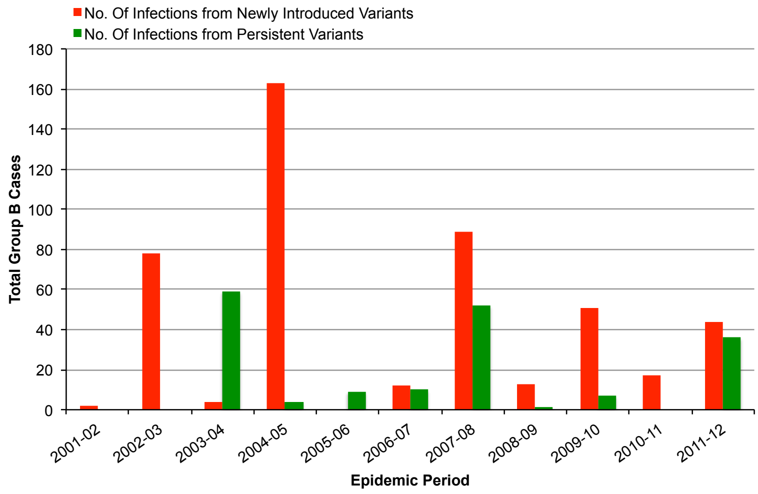

B

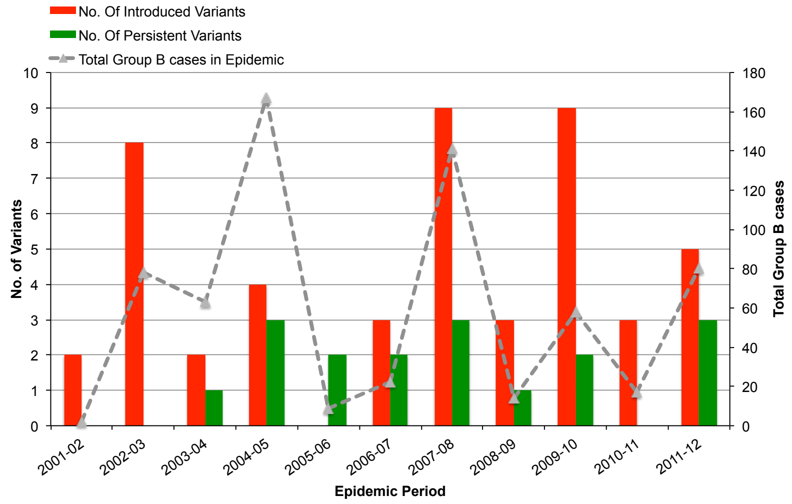

S6 Fig. Infection prevalence in relation to number of introduced versus persistent variants. **Panel A.** Frequency of infections from newly introduced variants versus persistent variants during each epidemic over the study period. Red bars indicate newly introduced variants while blue bars indicate persistent variants. **Panel B.** Changes in number of the group B introduced variants versus persistent variants relative to the total group B detections (secondary axis) during 11 epidemics that were observed in the surveillance. The number of variants from SAB1 and BA genotypes variants are combined**.**

S7. Fig. A BEAST tree showing the phylogenetic and temporal placement of the 911 unique Kilifi plus global sequences. The taxon names of Kenyan sequences are colored: red for Kilifi, blue for Nairobi, green for Dadaab and pink for Nyanza. Taxon names of sequences from outside Kenya (background diversity) are given in black. The node bars indicate the 95% HPD height interval while the size of the filled circles on the nodes indicate the posterior support for the associated branch. Note that for the global sequences that the date of sampling was given as year only, the exact date of sampling was assumed to 1st July. (next page)

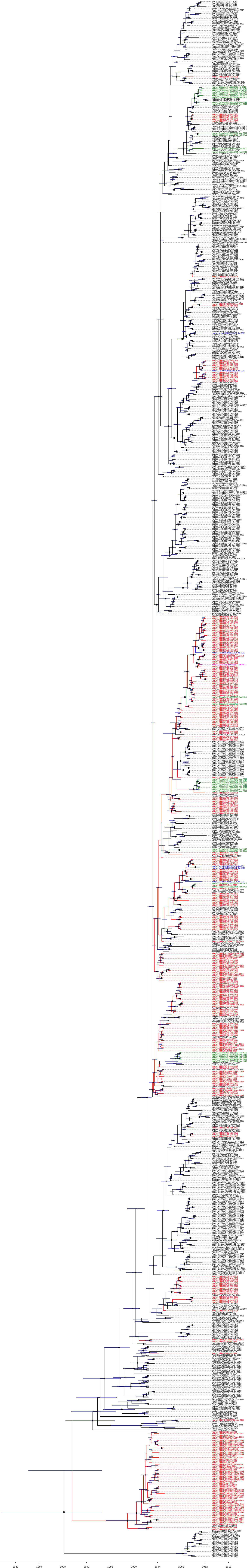
1980
1984
1988
1992
1996
2000
2004
2008
2012
2016
